# Integrating Non-Clinical Supports into Care: A Systematic Review of Social Prescribing Referral Pathways for Mental Health, Wellbeing, and Psychosocial Improvement

**DOI:** 10.5334/ijic.9127

**Published:** 2025-08-19

**Authors:** Samantha Spanos, Shalini Wijekulasuriya, Louise A. Ellis, Maree Saba, Tanja Schroeder, Charlotte Officer, Yvonne Zurynski

**Affiliations:** 1 Centre for Healthcare Resilience and Implementation Science, Australian Institute of Health Innovation, Macquarie University, Sydney, Australia; 2 Partnership Centre for Health System Sustainability, Australian Institute of Health Innovation, Macquarie University, Sydney, Australia; 3 Macquarie Medical School, Macquarie University, Sydney, Australia

**Keywords:** social prescribing, person-centred care, integrated care, mental health, wellbeing, psychosocial needs

## Abstract

**Introduction:**

Social prescribing (SP) involves person-centred integration of non-clinical supports into care, which is critical for addressing mental health and psychosocial needs and enhancing wellbeing. This systematic review evaluated the impact of SP referral pathways on mental health, wellbeing, and psychosocial outcomes.

**Methods:**

Four databases (Medline, Embase, PsycINFO and Scopus) were searched for empirical studies of referral pathways to community-based SP interventions targeting mental health, psychosocial factors and/or wellbeing. Data from studies published from January 2010 to April 2024 were extracted; referral pathways, interventions, and their resultant outcomes were synthesised and tabulated.

**Results:**

Across the 30 included studies, 27 SP interventions and two main referral pathways were identified, involving providers across health, community, social, and voluntary care sectors. Quantitative outcomes (*n* = 21, 70%) most frequently measured were wellbeing, anxiety, depression and quality of life. Findings were mixed, and widely varying methodological approaches limited study comparability. Qualitative outcomes (*n* = 16, 53%) were mostly associated with social interactions (e.g., increased sense of belonging) and self-concepts and feelings (e.g., increased sense of purpose).

**Discussion and conclusion:**

Research on SP referral pathways supporting mental health, wellbeing and psychosocial outcomes is proliferating, but high heterogeneity in the evidence base limits conclusive inferences about effectiveness. Standardised quantitative measurement of core outcomes, supplemented by rigorous qualitative designs, will enhance capacity to demonstrate the value of an SP approach to integrated care for mental and psychosocial health and wellbeing.

## Introduction

The escalating incidence of mental health conditions, and its associated surge in demand for mental health services, is a public health concern in many countries [1,2]. Primary threats to mental health and wellbeing globally include widening economic and social inequalities, public health emergencies like the COVID-19 pandemic, and the growing climate crisis [3,4]. An individual’s mental health directly influences their ability to cope with life stressors, succeed in learning and work, and contribute meaningfully to society, and is thus profoundly shaped by social, educational, and economic circumstances [4]. Mental health conditions frequently coexist with psychosocial problems, including issues with social functioning and participation, poor emotional support and wellbeing, and difficulties with employment and finances, requiring integrated, collaborative care across health, community, and social care sectors [5,6].

Social prescribing (SP) is a model of health and social care that involves connecting people with community resources to address unmet non-clinical needs [7,8]. Through its focus on person-centred care integration that promotes self-management and empowerment, SP is increasingly being recognised as a valuable model of integrated care for addressing mental health and psychosocial needs, improving wellbeing, and strengthening health system capacity [6,9,10,11,12]. SP can expand referral options for front line care providers to encompass non-clinical, community-based interventions [8,13], enabling providers to cater to individuals’ needs that are related to wider social determinants of health [6,14]. This supported referral focuses on the role of social engagement in health and wellbeing to address a broad spectrum of psychosocial needs (e.g., social, emotional, and environmental), and promotes holistic approaches to mental health beyond the traditional biomedical model [6,10,12,15]. A wide range of SP initiatives have been developed, encompassing arts, education, exercise and sports, nature-based activities, and supported access to specialised social and voluntary services (e.g., financial, housing, peer support) [6,7,8,16].

Emerging evidence highlights the positive impact of SP on health and wellbeing [17,18], alongside implementation and evaluation complexities due to diverse stakeholders and settings [13,19]. This has resulted in a lack of definitive evidence about the impact of SP, which is a key challenge to its uptake and endorsement [7,8,20,21]. A plethora of single-site SP studies exist that vary greatly in their methodologies, evaluations, and overall quality, limiting the extent to which outcomes can be compared and synthesised [17,18,22]. Less is known about the collective body of evidence for mental health, psychosocial and wellbeing outcomes specifically. There is substantial overlap in the measurement and categorisation of these outcomes [7,23,24], which warrants casting a wide net when examining the impact of SP on mental health. Prior reviews have been restricted to outcomes for specific intervention types (e.g., nature-based) [20,25,26] or specific healthcare settings (e.g., United Kingdom [UK]) [27], and there has been limited rigorous analysis of outcomes derived from qualitative research [20,22,28]. Given the well-recognised limitations of quantitative SP research (small scale, poor design, high risk of bias) [8,17,18,22], synthesising qualitative evaluations could provide valuable insights into the breadth of mental health, psychosocial and wellbeing outcomes, along with how and why they are impacted by SP [22,29].

Another gap in prior research is the lack of specific focus on outcomes for individuals who have engaged in SP via a referral pathway. Prior research has examined the impact of SP interventions for individuals recruited through a variety of avenues. Processes for identifying and referring people in need of mental health or psychosocial support are crucial for the success of SP as an integrated approach to mental health care; formal referral pathways are key to minimising care delays, identifying those who need care most, and reducing burden and confusion for healthcare providers [30,31,32]. Formal referral by a care provider can support better engagement in SP and better health outcomes [8,33], along with strengthening the continuity of care and the sustainability of community-based services coordinated around the needs of consumers [8,34,35]. There is a need for stronger evidence on the capacity of SP referral pathways to effectively deliver integrated care and services that address mental health and psychosocial needs and improve wellbeing [13,36].

The current review aims to synthesise the quantitative and qualitative literature on SP referral pathways that target mental health, psychosocial needs and wellbeing. In doing so, it aims to build on recent reviews of SP evaluation studies [7,23,24,37] to describe the breadth and range of mental health, wellbeing, and psychosocial outcomes of SP referral pathways, and evaluate the extent to which SP improves these outcomes.

## Research Methods

A systematic review of the peer reviewed literature was conducted and reported in accordance with the Preferred Reporting Items for Systematic Reviews and Meta-Analyses (PRISMA) [38]. This review was registered on the international prospective register of systematic reviews (PROSPERO; ID: CRD42024532873).

### Search strategy

A comprehensive search strategy was developed in conjunction with a medical librarian at Macquarie University, encompassing key terms related to SP (e.g., community referral, non-medical prescribing), mental health (e.g., anxiety, depression), psychosocial health (e.g., loneliness) and wellbeing (e.g., quality of life; see Appendix 1 for the search strategy). Medline, Embase, PsycINFO and Scopus were searched on 7 August 2023 for empirical articles published between January 2010 and August 2023. We searched publications in this date range to focus on contemporary SP approaches and evaluation methods, which are rapidly advancing [10,39]. The search was updated on 3 April 2024 covering the period August 2023 to April 2024. During screening, additional articles were also identified through backward citation searching (i.e., snowballing) of articles eligible for full-text review and of review articles.

### Study selection

All database search results were uploaded into Rayyan [40], an online data management platform, where duplicate records were identified and removed. Six reviewers (SS, SW, LAE, MS, TS, YZ) screened the articles according to the inclusion and exclusion criteria (Table 1). Studies were eligible if they reported on SP pathways that integrated community supports (inclusive of both participant-directed and pre-structured pathways), that involved referral by a care provider, and targeted mental health, psychosocial and/or wellbeing outcomes. To improve inter-rater reliability, 10% of the abstracts were screened by all reviewers (88% agreement in raw scores). Any disagreements or uncertainties were discussed by the team until consensus was reached. The full text of articles was screened by the same six reviewers using a data extraction form developed using the REDCap electronic data capture software hosted by Macquarie University. Five articles were independently full-text-screened by all reviewers to ensure consistency and usability of the extraction form, with the remaining articles apportioned among the reviewers and separately screened.

**Table 1:** Inclusion and exclusion criteria for study selection in systematic review.


Inclusion criteria:

Participants: Studies reporting on SP pathways targeted to adults (18+ years). Intervention: Studies reporting on SP pathways to community-based interventions, referred by care providers, and targeting mental health, wellbeing, or psychosocial outcomes.

Comparator: Studies comparing SP participants to a control group or evaluating longitudinal change in SP participants.

Outcomes: Studies reporting quantitative or qualitative outcomes related to mental health, wellbeing, or psychosocial health (before and after the intervention for quantitative studies).

**Exclusion criteria:**

Studies published prior to 1 January 2010.

Non-empirical studies (e.g., editorials, commentaries, letters) and conference abstracts.

No full-text available.

Not published in English.

Not set in a high-income OECD country.

Grey literature.


OECD, Organization for Economic Co-operation and Development; SP, social prescribing.

### Data extraction

Extracted data included details about the study (publication year, country, design, methods), participants (age, referral reason, symptoms), SP pathway (name, components, services, duration), referral provider and linkage process, quantitative outcomes (instruments, timepoints, sample sizes at each timepoint, type of analyses, reported direction of effect), control/comparison group (type, sample size), and qualitative outcomes (key themes and subthemes). Outcome data extracted were limited to mental health, wellbeing and psychosocial outcomes. Qualitative data were extracted using the thematic framework developed by Pescheny and colleagues [22]. This framework was chosen for data extraction and analysis because it was derived from a synthesis of studies closely aligned with the eligibility criteria of the current review.

### Data synthesis

Quantitative data were narratively synthesised through textual descriptions, groupings, tabulation and frequency analysis, using the ‘Guidance on the Conduct of Narrative Synthesis in Systematic Reviews’ [41]. Characteristics of included articles were summarised textually and using descriptive statistics. Details of referral pathways, interventions, participants, and outcome measurement were summarised and grouped into common categories, and the frequency of each category was calculated to determine the range and type of groupings across the included studies.

Qualitative data were analysed using the Framework Method approach [42]. Themes and subthemes were analysed using a combined deductive-inductive approach by three reviewers (SS, MS, TS), using the Pescheny et al. framework [22]. Reviewers familiarised themselves with the extracted qualitative data and used a combination of deductive and inductive techniques to identify and code, within each category of the Pescheny et al. framework, SP outcomes and their enablers. A thematic framework was created and iteratively revised as new codes and themes were identified. The stages of the data synthesis process are displayed in Appendix 2.

### Quality assessment

The Mixed-Methods Appraisal Tool (MMAT) [43] was utilised to assess the scope and quality of included studies. Two team members (SS, SW) assessed each study according to the appropriate MMAT criteria and resolved uncertainties through discussion, with a third team member available for arbitration if necessary. Discussions were focused on potential bias in study designs, analyses, and reporting, to determine whether each study adequately fulfilled the MMAT criteria. The studies that did not meet all assessment criteria were still included, as they provided valuable information about the quality of SP research.

## Results

### Search results

**Figure 1:**
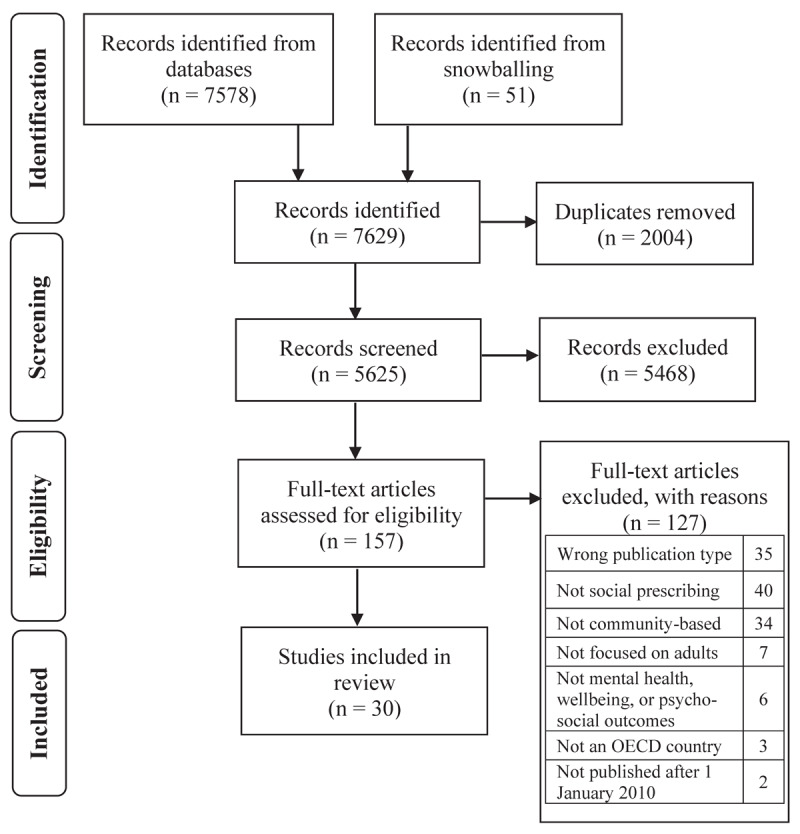
PRISMA flowchart displaying the process of identification and selection of included studies.

The study selection process is summarised in the PRISMA flow chart (Figure 1). Database searches yielded 7578 articles and snowballing yielded 51 articles. From a total of 7629 identified articles, 2004 duplicates articles were removed, and 5625 articles were screened by title and abstract. Following title and abstract screening, 5468 articles were excluded because they did not meet criteria or could not be retrieved, leaving 157 articles eligible for full-text review. Of these 157 articles, 127 were subsequently excluded for failing to meet the eligibility criteria, and the 30 remaining studies were included in the current review.

### Characteristics of included articles

Studies were conducted predominantly in the UK (*n =* 24, 80%), particularly England (*n =* 21, 70%). Two studies were conducted in Australia (7%), with the remaining four studies carried out in Sweden, Finland, Canada, and The Netherlands. Most studies (*n* = 18, 60%) had quantitative nonrandomised designs, which were either single-group longitudinal evaluations (*n* = 15) or matched comparison group evaluations (*n* = 3). Seven quantitative nonrandomised studies incorporated qualitative methodology (i.e., mixed methods; 23%). Nine studies (30%) were qualitative designs, and three studies (10%) were randomised controlled trials. To measure mental health-related outcomes of SP, studies primarily utilised quantitative surveys (*n =* 21, 70%) and interviews (*n =* 15, 50%); methods used less frequently were focus groups (*n =* 4, 13%), observations (*n =* 1, 3%), and patient diary entries (*n* = 1, 3%). The full list of included studies and their characteristics is provided in Appendix 3.

### Quality of included articles

Thirteen studies scored ‘yes’ responses across the relevant MMAT criteria, indicating high quality. Articles using quantitative nonrandomised designs (solely or combined with a qualitative design, *n* = 18) were typically uncontrolled before and after studies examining a single group. The most commonly failed criteria for these nonrandomised designs were appropriately adjusting for confounders in study design and analysis (*n* = 7), and providing adequate detail on whether the intervention was administered as intended (*n* = 7). For example, the delivery of some interventions were impacted by COVID-19 pandemic restrictions [44,45]. For the randomised controlled trial studies (*n* = 3), none reported that the outcome assessors were blinded to the intervention. Two studies that used mixed methods did not adequately address the divergences between qualitative and quantitative results [46,47]. Qualitative studies were generally of high quality (see Appendix 4).

### Target participants and reasons for referral

Most studies (*n =* 24, 80%) reported on SP that was targeted to adults in general with no specific age range. Four studies targeted older adults, with the minimum age of eligibility ranging from 50–65 years [48,49,50,51]. The primary reasons for referral were loneliness, social isolation, or unmet social needs (*n =* 14, 47%), poor mental health or wellbeing (*n =* 13, 43%), and the presence of chronic health conditions or risk of developing chronic conditions (*n =* 12, 40%). Other reasons for referral were bereavement/grief (*n =* 5, 17%), chronic pain (*n =* 5, 17%), psychosocial problems (*n =* 4, 13%), low self-esteem or confidence (*n =* 2, 7%), distraction from health-related issues (e.g., to support smoking cessation; *n =* 2, 7%), and cognitive issues (*n* = 1, 3%).

### Referral and linkage pathways

Almost all studies (*n =* 27, 90%) reported on SP referral through primary care. Primary care referrals were mostly initiated by general practitioners (*n =* 16, 53%) but also by practice nurses (*n =* 5, 17%), mental health professionals (*n =* 5, 17%), assistant practitioners (*n =* 2, 7%) and allied health professionals (*n =* 2, 7%). Studies also reported on referrals from the community sector (*n* = 7, 23%) third or voluntary sector (*n* = 5, 17%), secondary care (*n* = 4, 13%), social care (*n* = 4, 13%), and tertiary care (*n* = 2, 7%).

**Figure 2:**
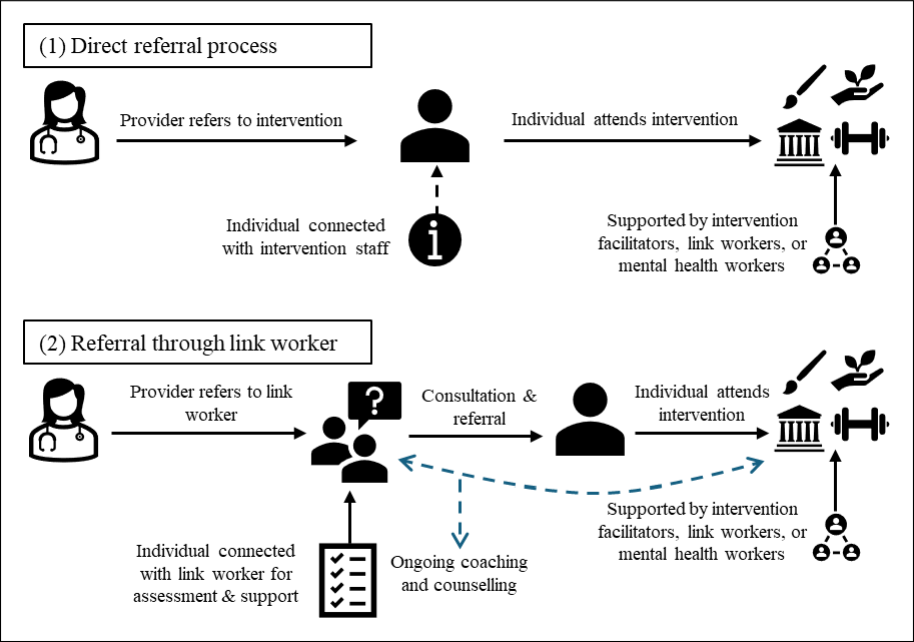
Two main referral and linkage pathways identified in the included studies.

The SP referral process varied among studies but was broadly categorised into two types: direct referral or referral through a link worker (Figure 2). The most common process was a direct referral from a care provider to an SP intervention (*n =* 21, 67%), where individuals were connected with intervention facilitators. In some of these direct referral pathways (*n* = 8), referral was followed by assessment and support by a link worker, mental health worker, or an expert intervention instructor (e.g., exercise professional) [46,51,52,53,54,55,56,57]. Referral from a care provider to a link worker was reported in 10 studies (33%), where participants were connected with a link worker for consultation and referral to a suitable intervention. Link workers were described with various terms including coordinator, navigator, wellbeing coach, community-links practitioner, or ‘holistic’ link worker, and terms were sometimes used interchangeably. Across both pathways, the SP process often involved ongoing coaching and counselling beyond the initial referral (*n =* 9, 30%) [48,54,56,58,59,60,61,62,63].

### Intervention types

Across 30 studies, 27 SP interventions targeting mental health, wellbeing, or psychosocial improvement were identified (see Appendix 3). Most common were arts-based interventions, 11 of which followed an Arts on Prescription model [64] (*n* = 13; 43%). Many arts-based interventions incorporated a broad range of activities including painting, drawing, pottery, photography, museum visits, theatre, music, and dance [44,47,49,55,65,66], and others focused on specific activities such as art-and-craft workshops [51,52,67,68] and museum visits [50,68]. Two studies provided little detail regarding the specific arts activities included in the intervention [64,69].

Tailored interventions were the second most common (9 interventions; *n* = 9, 30%) [48,53,57,58,59,60,61,62,63,70], in which a combination of activities was customised with a link worker to suit individual needs. These activities varied broadly and included arts, exercise, social groups, volunteering, and specific support groups. Intensive link worker support was typically provided as part of this tailoring process, in the form of coaching, motivational interviewing, action planning and progress monitoring [48,58,59,60,61,62,63].

Five interventions incorporated nature-based activities (*n* = 5, 17%), which focused on spending time in natural environments and included guided walks, learning about nature, and time for socialising [45,46,68,71,72]. Two of these interventions offered exercise as an alternative or adjunct to nature-based activities [45,46], and two studies described gardening-focused interventions such as creating a community garden [68,72].

Four interventions incorporated exercise activities (*n* = 4, 13%), such as walking, climbing, group sports and group exercise classes [45,46,54,56]. Two of these interventions involved intensive coaching and goal setting by a health or exercise professional [54,56]. Two interventions also offered nature-based activities as an alternative or adjunct to exercise [45,46].

### Quantitative evaluations

#### Types of evaluations

There were 21 quantitative evaluations conducted across all studies, with high variability in how outcomes were evaluated (see Appendix 5). Some studies examined outcome change over time within the same cohort of SP participants (i.e., within-group analyses), others examined change in SP participants compared to a comparison group (i.e., between-groups analyses), and some studies conducted both within-group and between-groups analyses. There was substantial variability in sample sizes utilised to measure change in outcomes and in the timepoints of outcome measurement. Studies that evaluated within-group differences in wellbeing had sample sizes ranging from eight to 651 participants, and post-intervention measurement timepoints ranged from five weeks to six months after intervention completion.

#### Outcomes, constructs and instruments

There were 10 outcomes measured that were relevant to the aims of the current review: wellbeing (*n* = 14), anxiety (*n* = 7), depression (*n* = 6), quality of life (QoL; *n* = 5), stress and psychological distress (*n* = 3), mood and affect (*n* = 3), loneliness (*n* = 3), belonging (*n* = 2), psychosocial needs and daily living (*n* = 2) and psychological energy (*n* = 1). Outcomes were measured differently across studies, in that different constructs or components within each outcome were examined. For example, four different constructs of wellbeing (e.g., mental wellbeing, museum wellbeing, health-related wellbeing, capability-based wellbeing) were measured. In total, 26 constructs were measured, and 25 different instruments were utilised to measure these constructs. The findings for each outcome are presented below.

*Wellbeing*. Wellbeing was the most commonly measured outcome (*n =* 14, 47%). Mental wellbeing was assessed using the Warwick Edinburgh Mental Wellbeing Scale (WEMWBS; *n =* 9) [45,47,48,49,65,67,71] or its shortened version (SWEMWBS; *n =* 1) [63]. Significant within-group improvement in mental wellbeing was demonstrated in eight studies [45,47,48,49,63,65,67,71], two of which reported clinically significant changes [48,71], but no significant between-group differences were reported [45,66]. Museum wellbeing was assessed using the University College London (UCL) Museum Wellbeing Measure (*n* = 2) and two studies demonstrated within-group improvement [50,68], with effect sizes reported in one study indicating clinically meaningful results [50]. Health-related wellbeing [58] and capability-based wellbeing [61] were also measured, but no significant improvements on these constructs were found.

*Anxiety*. Anxiety outcomes were measured in seven studies (23%). Anxiety symptoms were assessed using the anxiety subscale of the Hospital Anxiety Depression Scale (HADS-A; *n* = 5) [44,54,56,58,61]. Significant within-group improvement in anxiety symptoms was demonstrated in two studies [44,54], with one study reporting a large effect size [44]. Two studies demonstrated some significant between-group improvement in anxiety symptoms; for SP participants referred for mental health reasons [56], and for participants who engaged with a link worker three or more times [61]. Anxiety diagnosis was assessed using the Generalised Anxiety Disorder Scale (GAD-7; *n* = 2). Two studies showed significant within-group reduction in GADS-7 scores [65,71], but in one study scores were still aligned with a diagnosis of moderate anxiety [71].

*Depression*. Depression outcomes were measured in six studies (20%). Depression symptoms were assessed using the depression subscale of the Hospital Anxiety Depression Scale (HADS-D; *n* = 5) [44,54,56,58,61]. Significant within-group reduction in depression symptoms was found in two studies [44,54], with one study reporting a large effect size [44]. Significant between-group reduction in depression symptoms was demonstrated in three studies; one study showed improvement for all SP participants relative to a control group [44], and two studies showed improvement only for some SP participants (those who were referred for mental health reasons [56] and those who engaged with a link worker three or more times [61]). Depression diagnosis was assessed using the Patient Health Questionnaire (PHQ-8) in one study, which demonstrated significant within-group reduction in PHQ-8 scores [65].

*Quality of life*. QoL outcomes were measured in five studies (17%). Health-related QoL was assessed using the EuroQol-5D (*n* = 4) [52,56,61,70]. Significant within-group improvements were shown for overall health-related QoL [70] and on the health status rating scale of the EQ-5D [52]. Two studies demonstrated some significant between-group improvement in QoL; for SP participants aged 16–44 [56], and when SP participants engaged with a link worker three or more times [61]. Physical, psychological, social, and environmental QoL [52] and functional QoL [54] were also measured, and significant within-group improvement was demonstrated for physical, psychological and functional QoL.

*Stress and psychological distress*. Stress or psychological distress outcomes were measured in three studies (10%). Perceived stress, assessed using the Perceived Stress Scale (PSS), was shown to significantly decrease over time in SP participants [71]. Stress and crisis symptoms, assessed using the Stress and Crises Inventory-93 (SCI-93), also significantly decreased in SP participants over time, with a large effect size reported [44]. Psychological distress was also assessed, but no significant improvements on this construct was found [52].

*Mood and affect*. Mood and affect outcomes were measured in three studies (10%). Mood changes were assessed using a validated short mood scale [67], positive affect and negative affect were assessed using Positive and Negative Affect Schedule (PANAS-P and PANAS-N) [71], and negative states were assessed using the Dartmouth CO-OP Charts [54]. Within-group improvements in positive mood, positive and negative affect and negative states were demonstrated [54,67,71], as well as between-groups improvement in negative mood [54].

*Loneliness and social isolation*. Loneliness and social isolation outcomes were measured in three studies (10%). Two studies used the UCLA 8-item Loneliness Scale (ULS-8), and both found that SP participation was associated with significantly reduced loneliness over time [60,70]. One study assessed loneliness using the UCLA 3-item Loneliness Scale (ULS-3), but no significant improvements were found [52].

*Belonging*. Belonging outcomes were measured in two studies (7%). Both assessed group memberships using a list of social groups, and community belonging using a single item belonging scale [60,70]. Significant within-group increases in group membership and community belonging were associated with SP participation in both studies [60,70]. Social support was also measured in one study, but no significant associations with SP participation were found [70].

*Psychosocial needs and daily living*. Psychosocial needs and daily living outcomes were measured in two studies (7%). Health and social functioning was measured in one study using the Work and Social Adjustment Scale (WASAS) [61], and unmet health and social needs was measured in one study using the Camberwell Assessment of Need Short Appraisal Schedule (CANSAS) [52]. There were no significant differences reported on these constructs.

*Psychological energy*. Psychological energy outcomes were measured in one study (3%). Energy and vitality was measured using the Subjective Vitality Scale (SVS), and significant within-group (but not between-groups) improvement was demonstrated on this construct [54]. Psychological needs during exercise was measured to examine associations with health outcomes, but this relationship was beyond the scope of the current review [54].

### Qualitative outcomes

Qualitative outcomes of SP were reported in 16 studies (53%). Sample sizes ranged from six to 52 individuals, who were mostly interviewed immediately after completing the intervention, and only one study collected pre and post qualitative data [58]. Across 16 studies, 57 themes and eight subthemes were identified as relevant to the aims of this review, and SP outcomes and their enablers were identified within each category of the Pescheny et al. framework (see Appendix 6 for tabulated qualitative results).

#### Social interactions

Qualitative outcomes of SP were associated with social interactions in 15 studies (50%; 24 themes, two subthemes). Most commonly, outcomes of social interactions were an increased sense of belonging and community [46,49,51,53,55,57,64,68,71,72], enabled by feeling safe [47,55,64,72], supported by others [46,51,57], and connected to other SP participants in terms of shared experiences [49,51,57,64,68]. Feeling less alone was another common outcome of social interactions [47,53,57,60,62,68,71,72], enabled by feeling safe and supported and by having the opportunity to participate in shared activities [47,57,62,68]. Social interactions also contributed to increased confidence and personal growth [46,51,57,62,69,71], enabled through opportunities to connect with likeminded others and challenge perceptions of one’s abilities [57,69,71].

#### Self-concept and feelings

Qualitative outcomes of SP relating to self-concept and feelings were reported in 13 studies (43%; 23 themes, one subtheme). Most commonly reported changes included increased sense of purpose and meaning, enabled through feeling useful to others [53,59,68], motivated and accomplished [46,49,64,68,72], and connected to ‘something bigger’ [71,72]. Self-concept and feelings were also commonly related to increased confidence and self-esteem as a result of challenging one’s pre-set perceptions [47,51,57,69], learning new skills [64,68], and contributing to shared activities [55,68].

#### Health and wellbeing

Qualitative outcomes of SP relating to health and wellbeing were reported in 10 studies (33%; 15 themes, four subthemes). Health and wellbeing outcomes were most commonly related to improvements in mood, anxiety, emotional state and overall wellbeing, enabled by feeling supported and ‘seen’ [47,72], and by participating in activities involving exercise, relaxation and creativity [46,55,59,71]. For people with health conditions, improvements in condition management were reported, enabled by support for self-management, and increased physical activity and relaxation [71].

#### Health-related behaviours

Qualitative outcomes of SP relating to health-related behaviours were reported in 10 studies (33%; 16 themes, one subtheme). Health-related behaviours were most commonly associated with improved ability to initiate and implement actions to self-manage mental and physical health. These often involved the pursuit of new activities and hobbies for health improvement [46,49,51,57,69] and greater capability to set health-promoting goals [53,57,58,69]. Improved self-management behaviours were enabled by increased feelings of self-reliance and control [53,57,59,62,69], and motivation and confidence to explore new possibilities [46,49,51,57,58,69,71].

#### Day-to-day functioning

Outcomes of SP related to day-to-day functioning were reported in six studies (20%; six themes, one subtheme). Improved functioning was associated with increased motivation and ability to find employment, enabled by the development of life skills and increased confidence resulting from SP [55,58,64]. Increased motivation to seek and engage in further education was also reported, enabled primarily by increased confidence and support [55,64,69]. Additionally, ability to cope with day-to-day life was reported in three studies, enabled by support, feelings of safety, and structure provided by interventions [58,68,72].

## Discussion

This systematic review identified 30 empirical studies investigating the effectiveness of SP referral pathways for mental health, wellbeing, and psychosocial improvement. Most of these studies used quantitative nonrandomised designs to pilot the effectiveness of SP pathways on participant outcomes over time. Across the reviewed studies, SP referral pathways most frequently originated in primary care, and arts-based interventions were the most common endpoint of the pathways. Individuals with mental health needs were connected with interventions either directly by a care provider or via a link worker, and the linkage process often involved professionals from health, community, and social care sectors providing ongoing assessments and support to meet individual needs. Measuring the impact of care received via a range of delivery strategies, sectors and services is challenging [73]. SP referral pathways are highly variable, and the ways in which they are currently being evaluated limits what we can infer about their effectiveness on mental health, wellbeing, and psychosocial outcomes. We discuss this challenge below with reference to quantitative and qualitative outcomes.

### Quantitative outcomes

Consistent with the findings of previous reviews, this review revealed a great deal of heterogeneity in quantitative outcomes measured and tools used for measurement [18,22,37].

The most consistently assessed construct was mental wellbeing, measured using the WEMWBS, aligning with the findings of prior reviews [7,37]. In eight studies, mental wellbeing improved in SP participants over time, but these studies were uncontrolled, had widely varying and mostly limited sample sizes with minimal a priori power calculations, and measurement timeframes were narrow (as little as six weeks) [67,71]. Similarly, studies assessing anxiety, depression, and health-related QoL (which were the three other most consistently measured constructs) showed some improvement on these constructs over time, but these positive effects were not consistently demonstrated in between-groups evaluations [44,54], an issue identified in previous research [10]. The remaining 22 constructs were assessed in either one or two studies, most of which were uncontrolled (67%) and had inadequately powered sample sizes. These methodological limitations challenge the generalisability and reliability of the observed effects, which might have occurred for other reasons that were not accounted for (e.g., social desirability, lower scores at baseline) [10,17,36]. In the absence of longer-term follow-up, inferences cannot be made about the clinical meaningfulness of the findings.

Quantitative measurement of SP outcomes needs to be given a greater deal of thought by researchers. Individuals referred to SP often have complex problems related to the wider social determinants of health, and choice of measurement instruments must take this into account. Quantitative measurement of discrete constructs may not adequately capture change as individuals continue to face multifaceted life challenges [10]. While a wide range of quantitative measures could contribute to the evidence base for SP effectiveness, researchers should focus more on the intended effects of interventions and select appropriate measures that are responsive to change in the targeted populations [37]. There have been calls for standardised outcome measures aligned with the core aims of SP to build capacity for comprehensive evidence synthesis and informed decision-making across care contexts and in policy [8,21]. Frameworks for assessing integrated care performance and outcomes could be leveraged to develop an SP-specific assessment framework that incorporates key domains and indicators, with consumers as partners to ensure local relevance [73].

### Qualitative outcomes

Qualitative outcomes of mental health, wellbeing, or psychosocial improvement both reflected and differed from the observed quantitative outcomes captured in the included studies, and enabled greater understanding of *how* SP impacts mental and psychosocial health and wellbeing [22]. The most commonly reported qualitative outcome across studies was an increased sense of belonging and reduced loneliness, enabled by *social interactions*. This aligns with prior research which demonstrated that positive impacts of SP were largely driven by socialisation, and conceptualised positive social outcomes as uniquely different from health and wellbeing improvements [28]. Other research has conceptualised positive social outcomes as a component of improved health and wellbeing [74]. Our findings align with the former; different precursors and influencing factors were associated with belonging (e.g., feeling safe and supported [46,72]) than with wellbeing (e.g., improvements in mood and anxiety [47,72]). It is well recognised that the complex, multifaceted nature of wellbeing can make it difficult to comprehensively measure quantitatively [75,76], and qualitative studies can enhance understanding of the drivers of wellbeing, particularly the role of *belonging* and *connectedness* [77].

Other common qualitative outcomes reported were increased purpose and meaning, and increased confidence. Purpose and meaning was related to the *self-concepts and feelings* theme, and was enabled by feeling useful, motivated and accomplished. Increased confidence was both an outcome in its own right, enabled by various factors, and an enabler of outcomes. Although many SP approaches are designed to enhance empowerment and support meaningful, purposeful engagement [6,39], the quantitative instruments used for measurement appear lacking in their ability to capture these nuanced processes and outcomes [22]. The frequent measurement of wellbeing might be driven by the belief that improvements in wellbeing will then foster other outcomes, including improved confidence, self-esteem, and autonomy [39], but the directionality of this relationship can be called into question. Greater attention should be paid to how evaluations of SP for mental health can capture these nuanced outcomes. Rigorous qualitative methodologies with well-developed conceptual frameworks should be utilised in qualitative studies and as an adjunct to quantitative studies, to ensure that contextual factors influencing outcomes are identified and considered when interpreting results. Realist evaluations can be valuable for highlighting contextual factors that determine intervention success across different populations and settings [16,29].

### Strengths and limitations

This systematic review comprehensively synthesised and evaluated quantitative and qualitative research on SP referral pathways targeting mental health, psychosocial needs and wellbeing, canvassing the range of outcomes, measures, and evaluative approaches reported. The inclusion of qualitative research enabled a greater understanding of the varying person-centred integration approaches offered by SP, and the impacts these can have on mental health, and on social, emotional, and environmental needs that can influence mental health. Another strength of this review was the focus on research reporting outcomes of SP referral pathways, from the point of referral to intervention outcome measurement, rather than the outcome of SP interventions alone. By examining studies of SP referred by care providers, we can better understand the impact of integrating SP within healthcare systems.

There are limitations of this review worth noting. First, the design and conduct of this review did not involve people with lived experience accessing SP pathways. Nevertheless, the review provides a basis for future engagement with people with lived experience, enabling crucial insights into the measurement of outcomes during the co-design, implementation and evaluation of SP pathways targeting mental health. Second, it was outside the scope of this review to examine implementation evaluation, but we believe it to be the next key step for progressing research on SP as an integrated model of care for mental and psychosocial health. For example, it is essential to synthesise the research on the capacities of link workers and other interdisciplinary professionals required to enable effective SP referral pathways for individuals with mental health, psychosocial, and wellbeing needs [15]. Third, although our search strategy was comprehensively designed, we may have missed relevant studies reporting on similar models of care that leverage community resources to support individuals with mental health problems or psychosocial needs impacting their mental health [4]. Fourth, we did not search grey literature, which may contain relevant information about SP referral pathways that has not been indexed in academic databases. As a result, we may have missed other outcomes relevant to the aims of the current review. We did, however, include a broad range of evaluations (including uncontrolled studies) to ensure an accurate view of how SP is enacted in practice, but additional research that broadens these findings will be important, particularly in such a rapidly evolving field.

### Conclusion

Approaches to integrating non-clinical supports into care for people with mental health, wellbeing and psychosocial needs are increasingly being developed and evaluated. High heterogeneity in how SP referral pathways for mental health improvement are being evaluated limits conclusive inferences about their effectiveness. The development of SP referral pathways must be guided by specific goals and be accompanied by the design of rigorous mixed methods evaluation strategies. A standardised set of quantitative outcome measures alongside rigorous qualitative and implementation evaluations should be utilised to progress the evidence base on SP as a model of integrated care for holistic mental health improvement.

## Additional Files

The additional files for this article can be found as follows:

10.5334/ijic.9127.s1 Appendix 1. Search strings utilised for systematic review across databases.

10.5334/ijic.9127.s2 Appendix 2. Data synthesis process according to the framework method.

10.5334/ijic.9127.s3 Appendix 3. Characteristics of studies and social prescribing referral pathways for mental health improvement.

10.5334/ijic.9127.s4 Appendix 4. Quality assessment of included studies using the Mixed Methods Appraisal Tool.

10.5334/ijic.9127.s5 Appendix 5. Outcomes, constructs, and evaluation approaches across included studies.

10.5334/ijic.9127.s6 Appendix 6. Results of qualitative data synthesis guided by Pescheny et al. (2020).
