# Supplementary material for: Integrating Non-Clinical Supports into Care: A Systematic Review of Social Prescribing Referral Pathways for Mental Health, Wellbeing, and Psychosocial Improvement
Source: Int J Integr Care. 2025 Aug 19;25(3):21. doi: 10.5334/ijic.9127 (PMC12372674; doi:10.5334/ijic.9127)
Supplement: Appendix 1. — Search strings utilised for systematic review across databases. [file ijic-25-3-9127-s1.pdf]

Appendix 1. Search strings utilised for systematic review across databases

**MEDLINE**	
Social prescribing interventions	(“social prescri*” or “community prescri*” or "community refer*" or "link* scheme*" or “non-medic* prescri*”).mp<br>*OR*<br>((prescri* adj3 (green or blue or community or art or book or outdoor or forest or nature or animal or equi* or biblio*) and (therap* or intervention*)).mp
	AND
Mental health and wellbeing	Mental Health/ *OR*<br>exp Mental Disorders/ *OR*<br>exp Psychology/ *OR*<br>exp Psychiatry/ *OR*<br>exp Anxiety/ *OR*<br>Depression/ *OR*<br>exp Psychological Distress/ *OR*<br>Loneliness/ *OR*<br>exp Quality of life/ OR<br>(“mental health” or “mental illness*” or psychol* or psychiatr* or anxi* or depress*)
Limits	English language<br>1st Jan 2010 – 22 Aug 2023

**EMBASE**	
Social prescribing interventions	(“social prescri*” or “community prescri*” or "community refer*" or "link* scheme*" or “**non-medic* prescri***”)<br>*OR*<br>((prescri* adj3 (green or **blue** or community or art or book or outdoor or **forest or nature** or animal or equi* or biblio*) and (therap* or intervention*)).mp
	AND
Mental health and wellbeing	exp Mental Health/ *OR*<br>exp Mental Disease/ *OR*<br>exp Psychology/ *OR*<br>exp Psychiatry/ *OR*<br>exp Anxiety/ *OR*<br>exp Depression/ *OR*<br>exp Distress Syndrome/ *OR*<br>Loneliness/ *OR*<br>exp Quality of life/ OR<br>(“mental health” or “mental illness*” or psychol* or psychiatr* or anxi* or depress*)
Limits	English language<br>1st Jan 2010 – 22 Aug 2023

**PSYCINFO**	
Social prescribing interventions	(“social prescri*” or “community prescri*” or "community refer*" or "link* scheme*" or “non-medic* prescri*”)<br>*OR*<br>((prescri* adj3 (green or blue or community or art or book or outdoor or forest or nature or animal or equi* or biblio*) and (therap* or intervention*)).mp
	AND
Mental health and wellbeing	exp Mental Health/ *OR*<br>exp Mental Disorders/ *OR*<br>exp Psychology/ *OR*<br>exp Psychiatry/ *OR*<br>exp Anxiety/ *OR*<br>exp Depression (Emotion)/ *OR*<br>exp **Distress**/ *OR*<br>exp **Psychological stress** *OR*<br>exp **Quality of life**/ OR<br>**loneliness** *OR*<br>(“mental health” or “mental illness*” or psychol* or psychiatr* or anxi* or depress*)
Limits	English language<br>1st Jan 2010 – 22 Aug 2023

**SCOPUS**	
Social prescribing interventions	(“social prescri*” or “community prescri*” or "community refer*" or "link* scheme*" or “non-medic* prescri*”)<br>*OR*<br>((prescri* AND (green OR blue OR community OR art OR book OR outdoor OR forest OR nature OR animal OR equi* OR biblio*) AND (therap* OR intervention*))
	AND
Mental health and wellbeing	(“mental health” or “mental illness*” or psychol* or psychiatr* or anxi* or depress*)
Limits	English language<br>1st Jan 2010 – 22 Aug 2023

Note. Original search conducted on 22 August 2023. Search was updated using the same search strings on 3 April 2024.
