# Supplementary figures and images for: Integrating Non-Clinical Supports into Care: A Systematic Review of Social Prescribing Referral Pathways for Mental Health, Wellbeing, and Psychosocial Improvement
Source: Int J Integr Care. 2025 Aug 19;25(3):21. doi: 10.5334/ijic.9127 (PMC12372674; doi:10.5334/ijic.9127)

**Appendix 2.** Data synthesis process according to the framework method

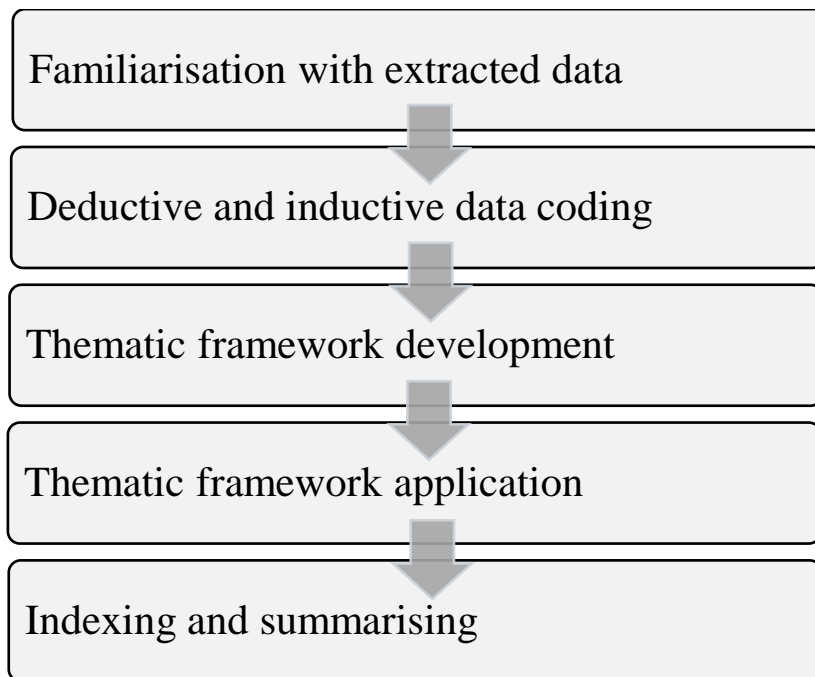

Supplement: Appendix 2. — Data synthesis process according to the framework method. [file ijic-25-3-9127-s2.pdf]
