# Supplementary material for: Integrating Non-Clinical Supports into Care: A Systematic Review of Social Prescribing Referral Pathways for Mental Health, Wellbeing, and Psychosocial Improvement
Source: Int J Integr Care. 2025 Aug 19;25(3):21. doi: 10.5334/ijic.9127 (PMC12372674; doi:10.5334/ijic.9127)
Supplement: Appendix 3. — Characteristics of studies and social prescribing referral pathways for mental health improvement. [file ijic-25-3-9127-s3.pdf]

Appendix 3. Characteristics of studies and social prescribing referral pathways for mental health improvement

Author (year)	Country	Study design	Referral pathway		Intervention		
			Referring providers or sectors*	Linkage process	Name or description	Type	Duration; intensity
Aggar (2021)	Australia	Quantitative longitudinal	GP	• Referral directly to intervention<br>• Assessment and support by mental health social worker	Plus Social Program	Arts	10 wks; 2-3h, weekly
Bergman (2023)	Sweden	Non-randomised controlled trial	Primary care, psychiatrist	• Signposting to intervention<br>• Stratified to intervention or control group by intervention instructor	Arts on Prescription	Arts	10 wks; 2.5h, twice weekly
Bhatti (2021)	Canada	Qualitative, descriptive	Community care	• Referral directly to intervention or to SP navigator<br>• Support to attend or become involved in intervention	Rx: Community-Social Prescribing	Tailored	NR
Carnes (2017)	England	Mixed methods non-randomised controlled trial	GP	• Referral to SP coordinator<br>• Consultation and referral to intervention by SP coordinator	Social prescribing coordinator	Tailored	6 sessions; ad hoc
Duda (2014)	England	Cluster-randomised controlled trial	GP, practice nurse	• Referral directly to intervention<br>• Assessment and support by health and fitness advisor	Exercise referral scheme	Exercise	12 wks; ad hoc
Elston (2019)	England	Quantitative longitudinal	GP, community care, social care, secondary care, tertiary care, third sector	• Referral to coordinator or holistic link worker<br>• Assessment, consultation and referral to intervention by link worker	Wellbeing Coordination Service	Tailored	12 wks; ad hoc
Heijnders (2018)	The Netherlands	Qualitative, descriptive	GP, assistant practitioner, physical therapist, psychologist	• Referral to wellbeing coach<br>• Consultation and referral to intervention by wellbeing coach	Welzijn op Recept	Tailored	Ad hoc
Holt (2020)	England	Quantitative longitudinal	Primary care	• Referral directly to intervention	Arts on Prescription	Arts	12 wks; 2h, weekly
Howarth (2021)	England	Qualitative, realist	Primary care, community care, SP connector	• Referral directly to intervention	Royal Horticultural Society Wellbeing Programme	Nature	12 wks; NR
Kellezi (2019, Study 1)	England	Qualitative, descriptive	GP, practice nurse	• Referral to link worker<br>• Assessment and support by health coach<br>• Consultation and referral to intervention by link worker	Social prescribing general practice referral pathway[a]	Tailored	8 wks; weekly or ad hoc
Kellezi (2019, Study 2)	England	Quantitative longitudinal	As above	As above	As above	As above	As above

Kolster (2023)	Finland	Non-randomised controlled trial	Primary care, social worker	• Referral directly to intervention<br>• Self-select intervention type	Int. 1: Health Forest project<br>Int. 2: Sport-based intervention	Nature / Exercise	8 wks; 7 sessions
Makanjuola (2023)	Wales	Mixed methods, longitudinal	GP, community mental health professional, third sector	• Referral directly to intervention<br>• Assessment and support by intervention instructor	Opening the Doors to the Outdoors programme	Nature / Exercise	12 wks; 4h, weekly
Makin (2012)	England	Qualitative, descriptive	GP	• Referral directly to intervention<br>• Assessment and support by mental health worker	Start 'Time Out' Arts on Prescription	Arts	6 mos; 2h, twice weekly
Maund (2019)	England	Mixed methods, longitudinal	Community mental health professional	• Referral directly to intervention	Wetlands for Wellbeing	Nature	6 wks; 2h, weekly
Mercer (2019)	Scotland	Cluster-randomised controlled trial	GP, practice nurse	• Referral to community links practitioner<br>• Consultation and referral to intervention by community links practitioner	Glasgow Deep End Links Worker Program	Tailored	Ad hoc
Moffatt (2017)	England	Qualitative, descriptive	GP, practice nurse, assistant practitioner	• Referral to link worker<br>• Consultation and referral to intervention by link worker	Ways to Wellness	Tailored	Ad hoc up to 2 years
Murphy (2012)	Wales	Randomised controlled trial	Primary care	• Referral directly to intervention<br>• Assessment and consultation with exercise professional	Wales National Exercise Referral Scheme	Exercise	16 wks; ad hoc
Payne (2020)	England	Qualitative, phenomenology	Primary care	• Referral directly to intervention<br>• Assessment and support by varying link workers	SOAR social prescribing	Tailored	Ad hoc
Pescheny (2021)	England	Quantitative longitudinal	GP	• Referral to link worker<br>• Consultation and referral to intervention by link worker	Luton social prescribing program	Tailored	12 sessions ad hoc
Poulos (2019)	Australia	Mixed methods, longitudinal	Range of medical providers, nurses, pharmacists, allied health, community care, pastoral carers	• Referral directly to intervention	Arts on Prescription	Arts	8-10 wks; weekly
Stickley (2012)	England	Qualitative, narrative	Primary care, mental health professional, third sector	• Referral directly to intervention	Arts on Prescription[b]	Arts	10 wks; NR
Stickley (2013)	England	Qualitative, narrative	Primary care, mental health professional, third sector	• Referral directly to intervention	Arts on Prescription[b]	Arts	10 wks; NR
Sumner (2019)	England	Retrospective cohort	Primary care, GP	• Referral directly to intervention	Arts on Prescription[c]	Arts	8-10 wks; weekly
Sumner (2021)	England	Retrospective	Primary care, GP, SP	• Referral directly to intervention	Arts on Prescription[c]	Arts	2 referrals of

		cohort	connector	• Self-select activity type			8 wks; weekly
Thomson (2018)	England	Quantitative longitudinal	Health care, social care, third sector	• Referral directly to intervention	Museums on Prescription	Arts	10 wks; 2h, weekly
Thomson (2020)	England	Mixed methods, sequential exploratory	Mental health nurse, community care	• Referral directly to intervention	GROW: Art, Park and Wellbeing	Arts / Nature	10 wks; 2h, weekly
van de Venter (2014)	England	Mixed methods, longitudinal	Primary care	• Referral directly to intervention	Arts on Prescription	Arts	20 wks; intensity NR
Vogelpoel (2014)	England	Mixed methods, longitudinal	GP	• Referral directly to intervention<br>• Assessment and support by intervention coordinator	Sense social prescribing	Arts	12 wks; weekly
Wakefield (2022)	England	Quantitative longitudinal	GP, practice nurse	• Referral to link worker<br>• Assessment and support by health coach<br>• Consultation and referral to intervention by link worker	Social prescribing general practice referral pathway[a]	Tailored	Ad hoc

GP, general practitioner; mos, months; NR, not reported; SP, social prescribing; wks, weeks.

[a, b, c] Articles examining the same social prescribing program are matched using superscript letters.
