# Supplementary material for: Integrating Non-Clinical Supports into Care: A Systematic Review of Social Prescribing Referral Pathways for Mental Health, Wellbeing, and Psychosocial Improvement
Source: Int J Integr Care. 2025 Aug 19;25(3):21. doi: 10.5334/ijic.9127 (PMC12372674; doi:10.5334/ijic.9127)
Supplement: Appendix 4. — Quality assessment of included studies using the Mixed Methods Appraisal Tool. [file ijic-25-3-9127-s4.pdf]

Appendix 4. Quality assessment of included studies using the Mixed Methods Appraisal Tool

Qualitative

First author	Year	S1. Are there clear research questions?	S2. Do the collected data allow to address the research questions?	1.1. Is the qualitative approach appropriate to answer the research question?	1.2. Are the qualitative data collection methods adequate to address the research question?	1.3. Are the findings adequately derived from the data?	1.4. Is the interpretation of results sufficiently substantiated by data?	1.5. Is there coherence between qualitative data sources, collection, analysis and interpretation?
Bhatti	2021	Yes	Yes	Yes	Yes	Yes	Yes	Yes
Carnes	2017	Yes	Yes	Yes	Yes	Yes	Yes	Yes
Heijnders	2018	Yes	Yes	Yes	Yes	Yes	Yes	Yes
Howarth	2021	Yes	Yes	Yes	Yes	Yes	Yes	Yes
Kellezi (study 1)	2019	Yes	Yes	Yes	Yes	Yes	Yes	Yes
Makanjuola	2023	Yes	Yes	Yes	No	Yes	Yes	Yes
Makin	2012	Yes	Yes	Yes	Yes	Yes	Yes	Yes
Maund	2019	Yes	Yes	Yes	Yes	Yes	Yes	Yes
Moffatt	2017	Yes	Yes	Yes	Yes	Yes	Yes	Yes
Payne	2020	Yes	Yes	Yes	Yes	Yes	Yes	Yes
Poulos	2019	Yes	Yes	Yes	Yes	Yes	Yes	Yes
Stickley	2013	Yes	Yes	Yes	Yes	Yes	Yes	Yes
Stickley	2012	Yes	Yes	Yes	Yes	Yes	Yes	Yes
Thomson	2020	Yes	Yes	Yes	Yes	Yes	Yes	Yes
van de Venter	2014	Yes	Yes	Yes	Yes	Yes	Yes	Yes
Vogelpoel	2014	Yes	Yes	Yes	Can't tell	Yes	Yes	Yes

Quantitative randomized controlled trials

**First author**	**Year**	**S1. Are there clear research questions?**	**S2. Do the collected data allow to address the research questions?**	**2.1. Is randomization appropriately performed?**	**2.2. Are the groups comparable at baseline?**	**2.3. Are there complete outcome data?**	**2.4. Are outcome assessors blinded to the intervention provided?**	**2.5 Did the participants adhere to the assigned intervention?**
**Duda**	**2014**	Yes	Yes	Yes	Yes	Yes	Can't tell	Yes
**Mercer**	**2019**	Yes	Yes	Yes	No	Yes	No	Yes
**Murphy**	**2012**	Yes	Yes	Yes	Yes	Yes	No	Yes

Quantitative non randomized

First author	Year	S1. Are there clear research questions?	S2. Do the collected data allow to address the research questions?	3.1. Are the participants representative of the target population?	3.2. Are measurements appropriate regarding both the outcome and intervention (or exposure)?	3.3. Are there complete outcome data?	3.4. Are the confounders accounted for in the design and analysis?	3.5. During the study period, is the intervention administered (or exposure occurred) as intended?
Aggar	2021	Yes	Yes	Yes	No	Yes	Yes	Yes
Bergman	2023	Yes	Yes	Yes	Yes	No	Yes	Yes
Carnes	2017	Yes	Yes	Yes	Yes	Yes	Yes	Yes
Elston	2019	Yes	Yes	Yes	Yes	Yes	Yes	Yes
Holt	2020	Yes	Yes	Yes	Yes	Yes	Yes	Yes
Kellezi (study 2)	2019	No	Yes	Yes	Yes	Yes	No	Yes
Kolster	2023	Yes	Yes	Yes	Yes	No	Yes	Yes
Makanjuola	2023	Yes	Yes	Yes	Yes	Yes	Yes	Yes
Maund	2019	Yes	Yes	Yes	No	Yes	Yes	Yes
Pescheny	2021	Yes	Yes	Yes	Can't tell	Can't tell	Yes	Yes
Poulos	2019	Yes	Yes	Yes	Yes	Yes	Yes	Yes
Sumner	2019	Yes	Yes	Yes	Can't tell	Can't tell	Yes	Yes
Sumner	2020	Yes	Yes	Yes	Yes	Can't tell	Yes	Yes
Thomson	2017	Yes	Yes	Can't tell	Can't tell	Can't tell	Yes	Yes
Thomson	2020	Can't tell	Yes	Yes	No	Yes	Can't tell	Yes
van de Venter	2014	Yes	Yes	Yes	Yes	Can't tell	Yes	Yes
Vogelpoel	2014	Yes	Yes	Yes	No	Yes	Yes	Yes
Wakefield	2022	Yes	Yes	Yes	Yes	Yes	Yes	Yes

Mixed methods

First author	Year	5.1. Is there an adequate rationale for using a mixed methods design to address the research question?	5.2. Are the different components of the study effectively integrated to answer the research question?	5.3. Are the outputs of the integration of qualitative and quantitative components adequately interpreted?	5.4. Are divergences and inconsistencies between quantitative and qualitative results adequately addressed?	5.5. Do the different components of the study adhere to the quality criteria of each tradition of the methods involved?
Carnes	2017	Yes	Yes	Yes	Yes	Yes
Makanjuola	2023	Yes	No	No	No	Yes
Maund	2019	Yes	Yes	Yes	Yes	Yes
Poulos	2019	Yes	Yes	Yes	Yes	Yes
Thomson	2020	Yes	Yes	Yes	Yes	Yes
van de Venter	2014	Yes	Yes	Yes	No	Yes
Vogelpoel	2014	Yes	Yes	Yes	Yes	Yes

Note. Each of these articles also has corresponding data for the relevant qualitative and quantitative components.
