# Supplementary material for: Integrating Non-Clinical Supports into Care: A Systematic Review of Social Prescribing Referral Pathways for Mental Health, Wellbeing, and Psychosocial Improvement
Source: Int J Integr Care. 2025 Aug 19;25(3):21. doi: 10.5334/ijic.9127 (PMC12372674; doi:10.5334/ijic.9127)
Supplement: Appendix 5. — Outcomes, constructs, and evaluation approaches across included studies. [file ijic-25-3-9127-s5.pdf]

Appendix 5. Outcomes, constructs, and evaluation approaches across included studies

Construct	Instrument	Author (year)	Within-group analyses: Measurement timepoints*	Within-group analyses: Sample size (n)	Within-group analyses: Sig. findings?	Between-groups analyses: Measurement timepoints*	Between-groups analyses: Sample sizes (n) Int.	Between-groups analyses: Sample sizes (n) Comp.	Between-groups analyses: Sig. findings?
WELLBEING (*n* = 14)									
Mental wellbeing	WEMWBS	Elston (2019)	6 and 12 mos.	86	Y	-	-	-	-
		Holt (2020)	6, 9 and 12 wks.	66	Y	-	-	-	-
		Kolster (2023)	8 wks.	54	Y	8 wks.	33	21	N
		Maund (2019)	6 wks.	16	Y	-	-	-	-
		Poulos (2019)	8-10 wks.	105	Y	-	-	-	-
		Sumner (2019)	10 wks.	651	N	10 wks.	651	607	N
		Sumner (2021)	8 wks.	96-245	Y	-	-	-	-
		Van de Venter (2014)	NR	44	Y	-	-	-	-
		Vogelpoel (2014)	12 wks.	8	N	-	-	-	-
	Short-WEMWBS	Pescheny (2021)	NR	63	Y	-	-	-	-
Museum wellbeing	UCL Museum Wellbeing Measure	Thomson (2018)	5 and 10 wks.	115	Y	-	-	-	-
		Thomson (2020)	10 wks.	20	Y	-	-	-	-
Capability-based wellbeing	ICECAP-A	Mercer (2019)	-	-	-	9 mos.	214-288	561-612	N
Health-related wellbeing	MYMOP	Carnes (2017)	8 mos.	65-184	N	8 mos.	65-184	126-300	N
ANXIETY (*n* = 7)									
Anxiety symptoms	HADS-A	Bergman (2023)	6 and 12 mos.	247	Y	6 and 12 mos.	247	232	N
		Carnes (2017)	8 mos.	65-184	N	8 mos.	65-184	126-300	N
		Duda (2014)	3 and 6 mos.	184	Y	3 and 6 mos.	184	163	N
		Mercer (2019)	-	-	-	9 mos.	214-288	561-612	Y[+]
		Murphy (2012)	-	-	-	12 mos.	479	513	Y[+]
Anxiety diagnosis	GAD-7	Maund (2019)	6 wks.	16	Y	-	-	-	-
		Sumner (2021)	8 wks.	96-245	Y	-	-	-	-

Construct	Instrument	Author (year)	Within-group analyses			Between-groups analyses			
			Measurement timepoints*	Sample size (n)	Sig. findings?	Measurement timepoints*	Sample sizes (n)		Sig. findings?
							Int.	Comp.	
DEPRESSION (*n* = 6)									
Depression symptoms	HADS-D	Bergman (2023)	6 and 12 mos.	247	Y	6 and 12 mos.	247	232	Y
		Carnes (2017)	8 mos.	65-184	N	8 mos.	65-184	126-300	N
		Duda (2014)	3 and 6 mos.	184	Y	3 and 6 mos.	184	163	N
		Mercer (2019)	-	-	-	9 mos.	214-288	561-612	Y^+
		Murphy (2012)	-	-	-	12 mos.	479	513	Y
Depression diagnosis	PHQ-8	Sumner (2021)	6 wks.	96-245	Y	-	-	-	-
QUALITY OF LIFE (*n* = 5)									
Health-related QoL	EuroQol	Aggar (2021)	6 mos.	10	N	-	-	-	-
		Mercer (2019)	-	-	-	9 mos.	214-288	561-612	Y^+
		Murphy (2012)	-	-	-	12 mos.	400	398	Y^+
		Wakefield (2022)	4 and 6-9 mos.	63-630	Y	-	-	-	-
Physical, psychological, social, environmental QoL	WHOQoL	Aggar (2021)	6 mos.	11	Y^+	-	-	-	-
Functional QoL	Dartmouth CO-OP domains	Duda (2014)	3 and 6 mos.	184	Y^+	3 and 6 mos.	184	163	N
STRESS AND PSYCHOLOGICAL DISTRESS (*n* = 3)									
Stress & crisis symptoms	SCI-93	Bergman (2023)	6 and 12 mos.	247	Y	6 and 12 mos.	247	232	N
Perception of stress	PSS	Maund (2019)	6 wks.	16	Y	-	-	-	-
Psychological distress	K10	Aggar (2021)	6 mos.	12	N	-	-	-	-
MOOD AND AFFECT (*n* = 3)									
Mood changes	Short Mood Scale	Holt (2020)	6, 9 and 12 wks.	66	Y	-	-	-	-
Positive affect	PANAS-P	Maund (2019)	6 wks.	16	Y	-	-	-	-
Negative affect	PANAS-N	Maund (2019)	6 wks.	16	Y	-	-	-	-
Negative states	Dartmouth CO-OP Charts	Duda (2014)	3 and 6 mos.	184	Y	3 and 6 mos.	184	163	Y

Construct	Instrument	Author (year)	Within-group analyses			Between-groups analyses			
			Measurement timepoints*	Sample size (n)	Sig. findings?	Measurement timepoints*	Sample sizes (n)		Sig. findings?
							Int.	Comp.	
LONELINESS (*n* = 3)									
Loneliness and social isolation	ULS-8	Kellezi (2019)	4 mos.	178	Y	-	-	-	-
		Wakefield (2022)	4 and 6-9 mos.	63-630	Y	-	-	-	-
	ULS-3	Aggar (2021)	6 mos.	11	N	-	-	-	-
BELONGING (*n* = 2)									
Group memberships	List of ten social groups	Kellezi (2019)	4 mos.	178	Y	-	-	-	-
		Wakefield (2022)	4 and 6-9 mos.	63-630	Y	-	-	-	-
Community belonging	Single item belonging scale	Kellezi (2019)	4 mos.	178	Y	-	-	-	-
		Wakefield (2022)	4 and 6-9 mos.	63-630	Y	-	-	-	-
Social support	Four-item social support scale	Wakefield (2022)	4 and 6-9 mos.	63-630	Y	-	-	-	-
PSYCHOSOCIAL NEEDS AND DAILY LIVING (*n* = 2)									
Health and social functioning	WASAS	Mercer (2019)	-	-	-	9 mos.	214-288	561-612	N
Unmet health and social needs	CANSAS	Aggar (2021)	6 mos.	11	N	-	-	-	-
PSYCHOLOGICAL ENERGY (*n* = 1)									
Energy and vitality	SVS	Duda (2014)	3 and 6 mos.	184	Y	3 and 6 mos.	184	163	N
Psychological needs during exercise	PNSES	Duda (2014)	3 and 6 mos.	184	-	3 and 6 mos.	184	163	-

CANSAS, Camberwell Assessment of Need Short Appraisal Schedule; Comp, comparison; CO-OP, COmprehensive OPtimal Performance; GADS-7, Generalised Anxiety Disorder Scale 7-item; HADS-A, Hospital Anxiety and Depression Scale – anxiety subscale; HADS-D, Hospital Anxiety and Depression Scale – depression subscale; ICECAP-A, Investigating Choice Experiments for the Preferences of Older People Capability Measure for Adults; Int, intervention; K10, Kessler Psychological Distress Scale; MYMOP, Measure Yourself Medical Outcome Profile; *n*, sample size; N, no; NR, not reported; PANAS-N, Positive and Negative Affect Schedule – negative affect subscale; PANAS-P, Positive and Negative Affect Schedule – positive affect subscale; PHQ-8, Patient Health Questionnaire 8-item; PNSEES, Psychological Need Satisfaction in Exercise Scale; PSS, Perceived Stress Scale; SCI-93, Stress and Crises Inventory – 93; SVS, Subjective Vitality Scale; UCL, University College London; WASAS, Work and Social Adjustment Scale; WEMWBS, Warwick Edinburgh Mental Well-being Scale; WHOWol, World Health Organisation Quality of Life; Y, yes.

*Timepoints of outcome measurement post-participation in SP intervention.

[+]Significant results were found for specific (but not all) groups of participants, referral reasons, timepoints, or interventions.
