# Supplementary material for: Integrating Non-Clinical Supports into Care: A Systematic Review of Social Prescribing Referral Pathways for Mental Health, Wellbeing, and Psychosocial Improvement
Source: Int J Integr Care. 2025 Aug 19;25(3):21. doi: 10.5334/ijic.9127 (PMC12372674; doi:10.5334/ijic.9127)
Supplement: Appendix 6. — Results of qualitative data synthesis guided by Pescheny et al. (2020). [file ijic-25-3-9127-s6.pdf]

Appendix 6. Results of qualitative data synthesis guided by Pescheny et al. (2020).

SOCIAL INTERACTIONS (n = 15)		SELF-CONCEPTS AND FEELINGS (n = 13)		HEALTH AND WELLBEING (n = 10)		HEALTH-RELATED BEHAVIOURS (n = 10)		DAY-TO-DAY FUNCTIONING (n = 6)	
**Outcomes**	**Enablers**	**Outcomes**	**Enablers**	**Outcomes**	**Enablers**	**Outcomes**	**Enablers**	**Outcomes**	**Enablers**
Increased sense of belonging	Feeling safe, supported and connected	Increased sense of purpose and meaning	Feeling useful, motivated and accomplished	Improved mood, anxiety, emotions and wellbeing	Feeling supported, participating in exercise, relaxation and creative activities	Improved self-management of health via pursuing new activities	Feelings of self-reliance and control, motivation and confidence to explore	Improved motivation and ability to find employment	Development of life skills, increased confidence
Reduced loneliness	Feeling safe and supported, opportunities to participate	Increased confidence and self-esteem	Challenging own perceptions, learning skills, contributing to shared activities	Improved health condition management	Support for self-management, increased physical activity and relaxation	Improved self-management of health via goal setting		Improved motivation to seek and engage in further education	Increased confidence and support
Increased confidence and personal growth	Opportunities to connect, challenging own perceptions							Ability to cope with day-to-day life	Feeling safe and supported, intervention structure
